# Group IIA secreted phospholipase A_2_ inhibition by elemolic acid as a function of anti-inflammatory activity

**DOI:** 10.1038/s41598-022-10950-1

**Published:** 2022-05-10

**Authors:** Aladahalli S. Giresha, Deepadarshan Urs, J. G. Manjunatha, P. Sophiya, B. H. Supreetha, Shankar Jayarama, K. K. Dharmappa

**Affiliations:** 1 Inflammation Research Laboratory, Department of Studies and Research in Biochemistry, Mangalore University, Jnana Kaveri Post Graduate campus, Chikka Aluvara, Kodagu, 571232 India; 2 Department of Chemistry, FMKMC College Madikeri, Mangalore University Constituent College, Mangalore, Karnataka 571201 India; 3 Department of Studies in Food Technology, Davangere University, Shivagangotri, Davangere, 577002 India

**Keywords:** Biochemistry, Biotechnology, Drug discovery, Immunology, Medical research

## Abstract

Human group IIA secreted phospholipase A2 (GIIA) is a key enzyme in inflammatory reactions, worsening the condition of several chronic inflammatory diseases. The natural inhibitors of GIIA potentially block the production of inflammatory mediators. In the present study, elemolic acid, a triterpenoid from *Boswellia serrata* inhibited the GIIA enzyme in a concentration-dependent manner with IC_50_ value of 5.70 ± 0.02 µM. The mode of GIIA inhibition was studied by increasing the concentration of the substrate from 30 to 120 nM, and calcium from 2.5 to 15 mM, the level of inhibition was not changed. The inhibitor-enzyme interaction was examined by fluorimetry and Circular Dichroism (CD) studies; elemolic acid altered intrinsic fluorescence intensity and shifted far UV- CD spectra of GIIA enzyme, suggesting the direct interaction with GIIA. Elemolic acid neutralized the GIIA mediated indirect hemolytic activity from 94.5 to 9.8% and reduced GIIA induced mouse paw edema from 171.75 to 113.68%. Elemolic acid also reduced the hemorrhagic effect of GIIA along with *Vipera russelii* neurotoxic non-enzymatic peptide -VNTx-II (VR-HC-I). Thus, the elemolic acid has been proven as a potent inhibitor of GIIA enzyme and modulated the GIIA induced inflammatory response by in situ and in vivo methods.

## Introduction

Inflammation is a pathophysiological process that involves a series of complex cascades of cellular and biochemical events that occur during tissue injury. Inflammation is a necessary prerequisite for lifesaving, but when it is prolonged results in detrimental implications like sepsis, systemic shock, and tissue injury^1^. Several scientific studies showed that human secreted phospholipase A_2_ enzymes play a role in many oxidative and inflammatory reactions^2^. Among the nine catalytically active human secreted sPLA_2_ enzymes, Group II secreted phospholipase A_2_ (GIIA) generally plays a significant role in causing chronic inflammatory diseases^3^. The GIIA concentration is usually meagre in healthy conditions (~ 3 ng/mL) but considerably increases during infections and inflammatory reactions (250–500 ng/mL)^4^. Supporting that, the raised GIIA concentration is evident in most of the inflammatory exudates and plasma of arthritis patients^5^, inflammatory bowel diseases, acute coronary syndrome^6^, asthma^7^, atherosclerosis^8^, acute respiratory distress syndrome (ARDS)^6^ and recently, elevated levels of GIIA was found in samples of COVID-19 patients and it is parallel to disease severity^9^. GIIA is also a biomarker for cardiovascular diseases^10,11^, sepsis^12^ and chronic graft failure^13^.

**Figure 1:**
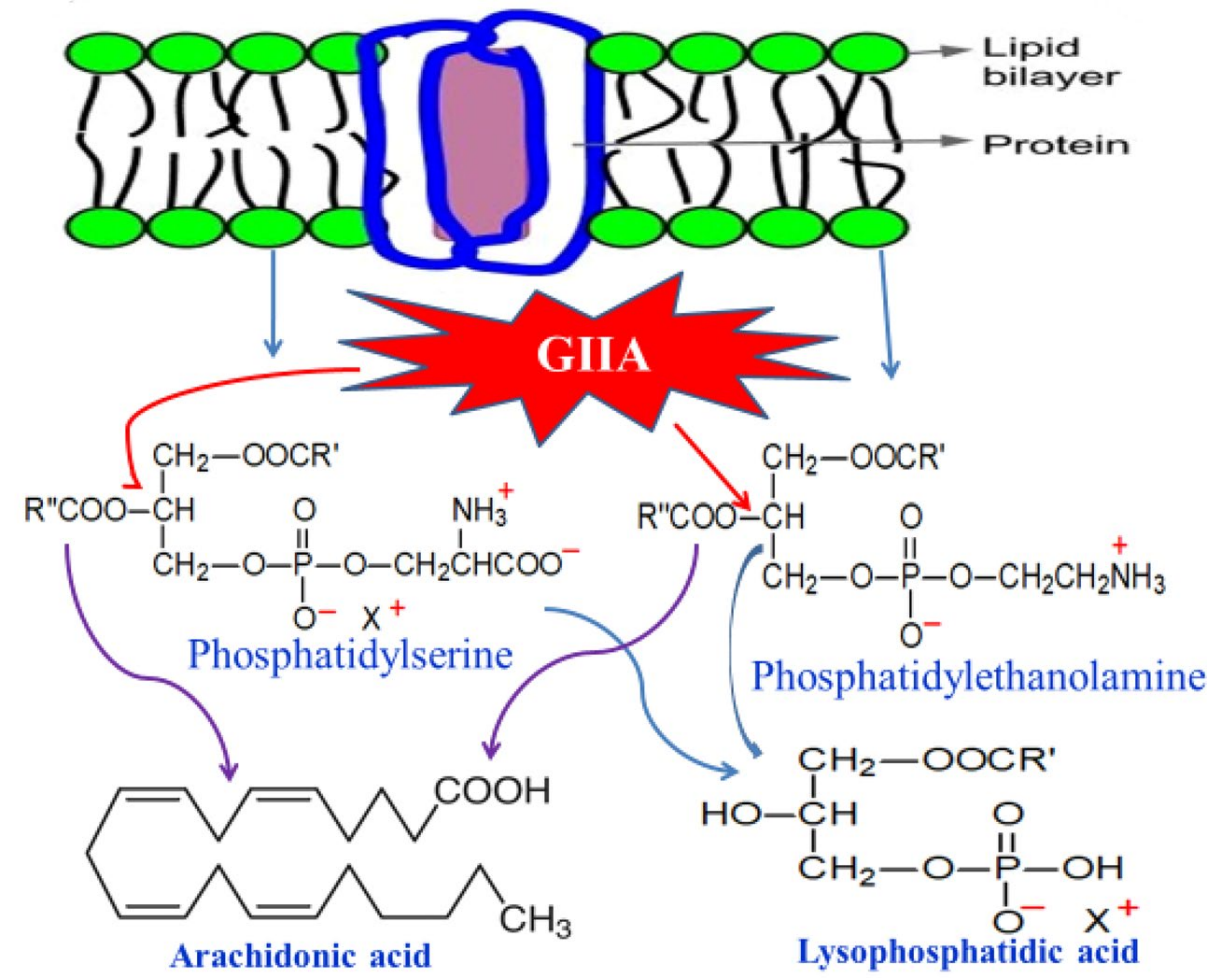
Schematic representation of GIIA catalytic hydrolysis of membrane phospholipids into arachidonic acid and lysophosphatidic acid.

GIIA catalyzes the hydrolysis of phospholipid substrates such as phosphatidylethanolamine (PE), phosphatidylserine (PS), phosphatidylglycerol (PG)^9^ and phosphotidylcholine into arachidonic acid (AA) and lysophosphatidate (Fig. 1). The arachidonic acid catalyzed by cyclooxygenase-1/2 (COX-1/2) and lipoxygenase (LOX) enzymes into pro-inflammatory eicosanoids such as prostaglandins, thromboxanes, prostacyclins, and leukotrienes respectively. Another GIIA product, lysophosphatidic acid (lysophosphotidylcholine) is catalyzed by acetyltransferase into platelet activation factor (PAF)^14^ that continues to cause inflammation by activating neutrophils and mast cells.

GIIA produces a lot of Reactive Oxygen Species (ROS) through LOX and COX-generated metabolites by stimulating NADPH oxidases^15^ and modulates the activity and function of cPLA_2_ and iPLA_2_ for the significant increase in arachidonic acid metabolism, which in turn contribute to the production of free radicals^16^. Interestingly, ROS in turn activates GIIA or may enhance membrane lipid peroxidation, which in turn brings about all downstream reactions, thereby profoundly increase the concentration of inflammatory mediators, result in inflammatory diseases. Hence, a molecule with both the antioxidant activity and GIIA inhibitory property is advantageous to becoming an anti-inflammatory molecule.

Since a long ago, Non-Steroidal Anti-Inflammatory Drugs (NSAIDs) have been considered for treating inflammatory diseases^17,18^. NSAIDs inhibit cyclooxygenase COX-1 and COX-2; and do not effect on leukotrienes or PAF^19^. Prolonged use of these drugs leftover various side effects such as hepatotoxicity, cardiovascular complications, and gastrointestinal toxicity^20–22^. In recent years, specific GIIA inhibitors varespladib (LY315920) and varespladib methyl (LY333013) are studied in clinical trials^23^. Also, a few potent drug candidates such as ginkgetin, petrosaspongiolide M, manoalide, and cacospongionolide B are evaluated in phase II clinical trials^24,25^. Unluckily, all the drugs failed to enter the market as effective anti-inflammatory drugs even though they inhibit GIIA in very low concentration, mainly due to the problems associated with formulation and their cytotoxic nature. Hence, there is a due urge in the concerned field for the safe and potent GIIA inhibitor as an anti-inflammatory molecule.

The resin of *Boswellia serrata* (*B. serrata*) was used in Indian classical medicinal system to treat a various of inflammatory conditions that affects the eyes, skin, gums, gastrointestinal tract (GIT), and the disorders like rheumatoid arthritis^26^, bronchial asthma^27^, chronic colitis^28^ and Crohn’s disease^29^. Oral consumption of *B. serrata* gum resin results in reduced levels of inflammatory mediators such as TNF-α, IFN-γ, IL-1β, IL-6, and PGE2 in rats^30^. The safety, efficacy, and tolerability of *B. serrata* extract were examined in 66 osteoarthritis patients for six months. The result was compared with a COX‐2 inhibitor valdecoxib, which revealed that the *B. serrata* extract was superior to valdecoxib, and very few individuals experienced acidity, abdominal cramps, and diarrhea^31^. The European Medicines Agency (2002) classified *B. serrata* extract as an ‘orphan drug’ to treat peritumoral brain edema^32^.

The objective of the present study is to discover safe and potent natural bioactive molecule/s from *B. serrata* for GIIA inhibition. Many triterpenoids such as oleanolic acid^33^, celastrol^34^, maslinic acid^35^, and ursolic acid^36^ are well documented for GIIA inhibition. Hence, a triterpenoid, elemolic acid was identified by molecular docking study and employed to validate the anti-inflammatory and pharmacological activities of *B. serrata* extracts. Hence, elemolic acid (IC_50_ value of 5.70 ± 0.02 µM) was subjected to GIIA inhibition by *in-vitro, in-situ, *in vivo methods and tested its antioxidant activity.

## Materials and methods

### Chemicals and reagents

Procured elemolic acid, gallic acid, 2, 2-diphenyl-1-picrylhydrazyl radical (DPPH˙), thiobarbituric acid (TBA), Sephadex (G-25, 50 and 75), CM-Sephadex C-25, Scintillation cocktail (Ultima Gold) and dimethyl sulfoxide (DMSO) from Sigma-Aldrich (St. Louis, MO, USA). ^14^C-oleic acid was purchased from Perkin Elmer Life Sciences Inc. Boston, USA. The *Vipera russelii* venom was obtained from Irula Cooperative Society Ltd., Chennai, India. All other chemicals and reagents used in this study were in higher quality.

### Animals

The study was conducted according toARRIVE guidelines. Swiss albino mice (male; 20–25 g) were obtained at eight weeks from the Animal House Facility (AHF), Department of Studies in Zoology, Mangalore University, India. Animals were acclimatizatised by exposing them to 12-h light and 12-h dark cycles, temperature of 22–25 °C, and 40% humidity conditions with free access to standard diet and autoclaved water. Then, the mice were individually housed in autoclaved cages and sterile hardwood chip bedding. Experiments were conducted as per the protocols of Institutional Animal Ethical Committee (IAEC), Mangalore University India (No: MU/AZ/504(a)/IAEC/2015-2016)^37^_._

### Human biological fluid

The Institutional Human Ethical Committee permitted the usage of human blood samples (IHEC), Mangalore University, Mangalore, India (IHEC-No.MU/IHEC/2018/7). The study was performed in according to the Helsinki recommendations and, institutional guidelines and regulations. The informed consent was obtained from healthy volunteers prior to the commencement of the study.

### Purification of GIIA

The GIIA enzyme was purified from *Vipera russelii* venom as described by Kasturi et al*.*^38^. Homogeneity of GIIA was checked by SDS-PAGE^39^. The phospholipase A_2_ (GIIA) of *Vipera russelii* venom belongs to group IIA generally used to reveal the mode of action of human inflammatory GIIA and inhibition studies because of its availability, simple purification procedures, high degree of structural similarities, and catalytic activity to human sPLA2^40^.

### Molecular docking

Autodock vina 1.1.2 in PyRx 0.8 was used to conduct a molecular docking study^41^. The crystal structure of secreted human inflammatory phospholipase A_2_ (GIIA) was taken from the protein data bank (PDB id: 1POE), and the structure of phytoconstituents in 3D SDF format was retrieved from PubChem structure database^42^_._ GIIA and phytochemicals structures were developed and docked on a grid, and the center value was 28.3252 X 3.8846 X 69.9194. In the docking study, the drug molecule was flexible, and GIIA was inflexible. For the optimal conformation, the molecule with the lowest binding score with the highest binding affinity was selected^43^. PoseView (version 1.1.2) was used to examine the interactions of different GIIA residues with inhibitors via hydrogen bonds, hydrophobic interactions, and electrostatic interactions.

### Estimation of in vitro antioxidant activities

The in vitro antioxidant activity of elemolic acid was estimated as per the method of Blois^44^. Briefly, 25 μM of elemolic acid was added to methanolic DPPH solution (0.01 mM) and incubated for 20 min in the dark condition. Then optical density was measured at 517 nm. DPPH alone served as a positive control, and ascorbic acid was the standard.

Anti-lipid peroxidation assay was performed as per the method of Gutteridge^45^. Briefly, the lipid peroxidation was induced by adding FeCl_3_ (7 mM) to the solution containing egg homogenate (10%) with 25 μM elemolic acid and incubated for 30 min. The reaction was ended by adding 1 mL of TBA (0.8%) and TCA (20%). The reaction without elemolic acid served as a positive control, and alpha-lipoic acid was taken as standard. Lipid hydroperoxides released were extracted with butanol and estimated by reading optical density at 530 nm. The antioxidant activities of the above methods are expressed using the following formula,

$$\%\,Antioxidant\,\,activity=\frac{\left(Control\,\,O.D\,-\,Sample\,\,O.D\right)}{Control\,\,O.D}X100$$

The reducing power assay was carried out as per the method of Oyaizu^46^. 25 μM of elemolic acid was mixed with 490 μL of phosphate buffer of 0.2 M (pH 6.6) and 500 μL of potassium ferricyanide (1%), which was incubated for 20 min at 50° C. The reaction ended by adding 0.5 mL of TCA (10%), and the reaction mixture was centrifuged at 3500×*g* for 10 min. 1 mL of supernatant was collected and mixed with 1 mL of distilled water, added 0.1 mL of 0.1% FeCl_3_ to develop a colored complex which was read at 700 nm. An increase in absorbance compared to control indicated the ferric reducing antioxidant power. The reaction mixture without standard or test served as a blank. We calculated the percent increase in reducing power using the following equation.

$$\% \,Reducing\,\,power = \frac{{\left( {Test\,\,O.D~ - ~Blank\,\,O.D} \right)}}{{Blank\,\,O.D}} \times 100$$

### Secreted phospholipase A_2_ assay (GIIA)

The GIIA enzyme activity was estimated by using autoclaved *E. coli* labeled with ^14^C-oleic acid according to Patriarca et al.^47^ and the modified method of Vishwanath et al*.*^48^. Briefly, a 350 µL reaction mixture consisting of 3.18 × 10^9^ autoclaved *E. coli* cells (corresponds to 10,000 cpm and 60 nmol lipid phosphorus), 5 mM calcium (CaCl_2_), and 100 mM Tris–HCl buffer pH 7.4 were mixed in the following order, buffer, calcium, enzyme (20 µg), water. Finally, added *E. coli* substrate (30 µL) and incubated at 37 °C for 60 min. Adding 100 µL of 2 N HCl and 100 μL fatty acid free BSA (10%) to terminate the reaction, vortexed the reaction mixture and centrifuged at 20,000×*g* for 5 min. 140 µL supernatant (containing ^14^C-oleic acid) was carefully collected, and added scintillation cocktail and measured the radiation of ^14^C using a Quantulus 1220 liquid scintillation spectrometer (Perkin Elmer, USA). GIIA activity was expressed as nmol of free fatty acid (^14^C-oleate) released/min/mg of protein under standard conditions.

### Inhibition of GIIA activity

The 10 mg elemolic acid was dissolved in 1 mL DMSO and made up to the appropriate concentration with the Tris–HCl buffer. GIIA inhibition was carried out with indicated concentrations of elemolic acid in the range of 2 to 16 µM. The previous report showed that genistein is a promising inhibitor of GIIA proven as an anti-inflammatory molecule by in vitro, in situ, and in vivo (reduced the mouse paw edema) experiments, used as a positive control. The highest concentration of DMSO used was 0.022 percent. The GraphPad Prism Version 5.0, USA software was used to calculate IC_50_ value.

### Effect of calcium and substrate concentration on GIIA inhibition

The effect of calcium and substrate concentrations on GIIA inhibition was studied. In separate assays, GIIA activity was measured by increasing the concentration of calcium from 2.5 to 15 mM, and substrate from 30 to 120 nmol in the presence and absence of IC_50_ concentration of elemolic acid (5.70 µM), and the assay was carried out as stated above.

### Determination of binding characteristics and reversibility of GIIA inhibition

In this study, GIIA enzyme was pre-incubated with IC_50_ concentration of elemolic acid (5.70 µM) in a 350 µL reaction mixture and dialyzed (MW cut off—3,000–6,000) for twenty-four hours with two buffer changes. GIIA activity was measured before and after the dialysis.

### Intrinsic fluorescence interaction study

The intrinsic fluorescence intensity of GIIA enzyme with and without elemolic acid was measured in Horiba JobinYvonFluorolog—3 spectrofluorometer (Centre of excellence and Nano Science (CeNS, Bangalore, India). The 2.0 mL reaction mixture in quartz cuvette of 1 cm path length consists, GIIA (20 µg/mL), 100 mM Tris–HCl buffer (pH 7.4), 5 mM calcium and increasing concentrations of elemolic acid (0.02 to 0.10 µM). The fluorescence spectra was measured between 300 and 370 nm after the excitation at 280 nm. Due to the internal absorption and filtration, the elemolic acid caused the quenching of spectra non-specifically. The tryptophan standard was used to correct it empirically^49^. The spectra for blank containing 100 mM Tris–HCl buffer (pH 7.4), 5 mM calcium, and 0.02% DMSO were substracted from spectra of GIIA and GIIA with different concentrations of elemolic acid.

### Circular dichroism study

Far UV-CD spectrum was recorded for GIIA enzyme (30 µg/mL) with or without elemolic acid (IC_50_ concentration, 5.70 µM) in a standard reaction mixture using Jasco J-810 spectropolarimeter at the Centre of excellence and Nano science (CeNS), Bangalore, India. The spectra was obtained using a quartz cuvette with a path length of 1 cm between 200 and 240 nm at room temperature. The response time was 2 s and the bandwidth was 1 nm. A total of ten scans were used to get the final spectra. Spectrum of blank contained Tris–HCl buffer (100 mM), 5 mM calcium, and DMSO (0.022%) and was subtracted to correct the protein spectra. K2D3 software was used to calculate the secondary structure of GIIA using CD spectral data.

### Neutralization of GIIA induced indirect hemolytic activity

The assay was carried out as per the method of Boman and Kaletta^50^. The substrate was prepared by mixing freshly packed human RBC (1 mL) and egg yolk (1 mL) in 8 mL of PBS. GIIA (30 µg) was pre-incubated with elemolic acid in the range of 2 to 16 µM at 37 °C for 30 min. Then, 1 mL of the substrate was added to this pre-incubated reaction mixture and incubated at 37 °C for 45 min. Halted the reaction by adding 9 mL of ice-cold PBS and centrifuged it for 20 min at 1500×*g*. The hemolytic activity in terms of released hemoglobin was measured at 530 nm. GIIA enzyme without corosolic acid in the sample served as a positive control.

### Neutralization of edema inducing activity

The method of Yamakawa et al.^51^, adapted by Vishwanath et al.^52^ was followed. The GIIA (5 µg) alone or with different concentrations of elemolic acid (3 to18 µM) in a total volume of 20 µL was injected into the intraplantar surface of the right hind footpad of mice weighing 20 to 25 g. 20 µL saline was injected into the respective left footpad for control. The animals were euthanized after 45 min by administering anesthesia (30 mg/kg of pentobarbital i.p.), and both the hind limbs were cut at the ankle joint and weighed separately. The percentage of edema was calculated by the following formula,

$$Edema\,\,ratio = \frac{{{\text{Weight}}\,{\text{of}}\,{\text{the}}\,{\text{edematous}}\,{\text{leg}}}}{{{\text{Weight}}\,{\text{of}}\,{\text{normal}}\,{\text{leg}}\,\left( {{\text{saline}}\,{\text{injected}}} \right)}} \times 100$$

### Neutralization of hemorrhagic activity of GIIA was estimation

The method of Kondo et al.^53^, modified by Venkatesh et al.^54^ was used to determine hemorrhagic activity. Briefly, mice were injected 10 μg of hemorrhagic complex containing 5:2 ratio of GIIA enzyme and non-enzymatic peptide (Vipera neurotoxin-II VNTx-II) subcutaneously (s.c). For the inhibition study, the hemorrhagic complex was pre-incubated with indicated concentrations of elemolic acid (5 µM, 10 µM, and 15 µM) for 30 min. Saline alone served as a negative control. After three hours, the mice were euthanized by administering pentobarbital (30 mg/kg, i.p.) and sacrificed by cervical dislocation. The skin was removed and hemorrhagic spots on the dorsal surface of the skin were measured using graph sheet. The results were expressed in mm^2^ of hemorrhagic spots.

### Statistical analysis

The experimental results were reported as the mean ± SD of three determinations. Graph Pad prism version 5.0 was used to calculate the IC_50_ values and dissociation constant (KD) (La Jolla, USA) and calculated the percentage of inhibition from the difference between animals of inhibitor-treated and control that received the vehicle.

### Ethics approval

All experiments were performed in accordance with AVMA guidelines^55^. Animal experiments were performed after obtaining animal ethical approval (No: MU/AZ/504(a)/IAEC/2015–2016) and human blood sample was collected from healthy volunteers after obtaining ethical approval and informed consent letter (IHEC-No.MU/IHEC/2018/7).

## Result and discussion

**Figure 2:**
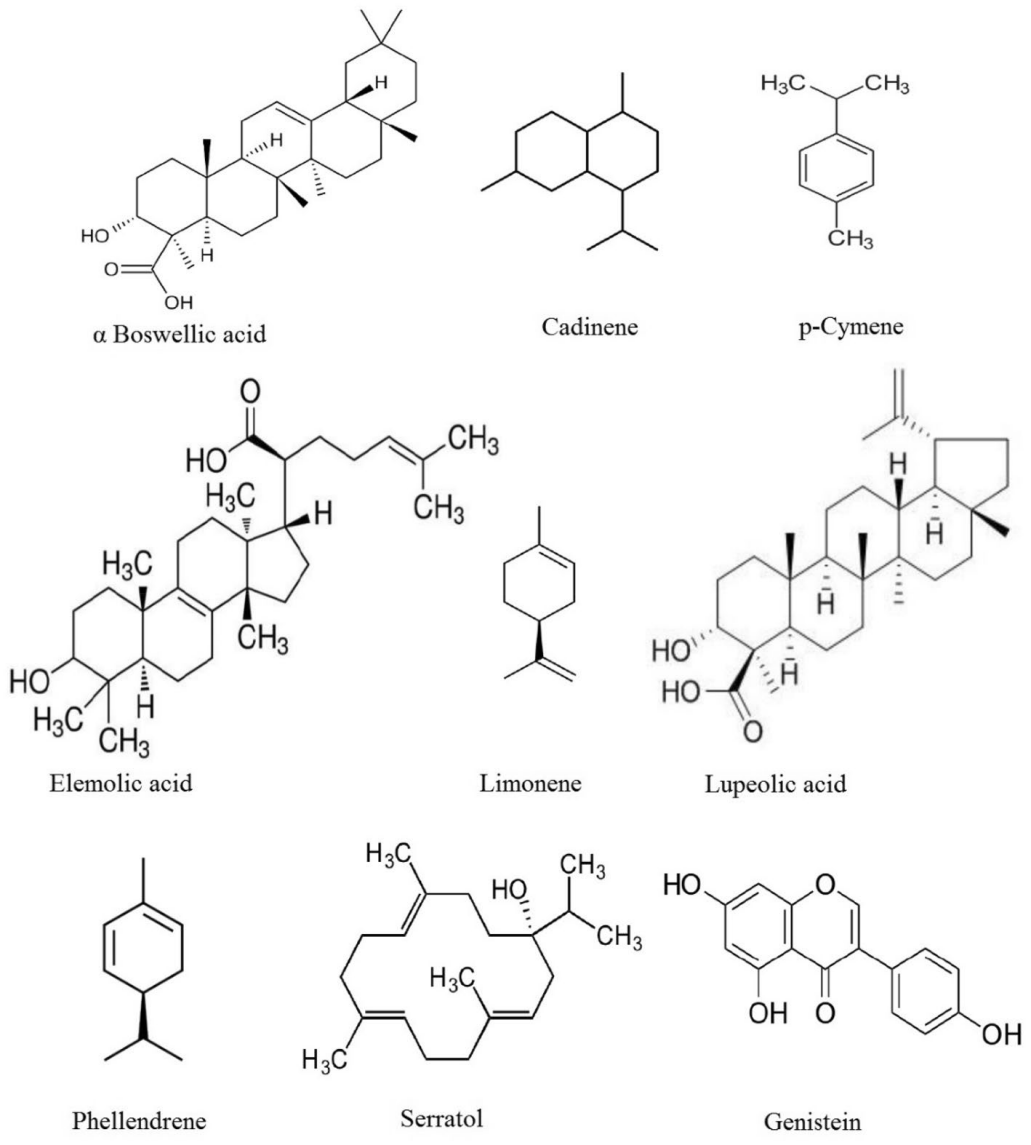
Structures of bioactive compounds from *B. serrata.*

The previous studies reported that the methanolic extract of *B. serrata* contains the bioactive molecules such as monoterpenoids (phellendrene, cadinene, limonene, p-cymene), diterpenoid (serratol) and triterpenoids (lupeolic acid, elemolic acid, α Boswellic acid, acetyl-α-boswellic acid) exhibited several pharmacological activities^56–58^(Fig. 2). These molecules were subjected to in silico docking study for identification of potent GIIA inhibitor/s. The docking study with respect to enzyme-inhibitor binding affinity was exploited and expressed as energy-value (E-value). The energy values (E values) denote binding energy. A greater negative E*-*value indicates a strong interaction with receptors. If the binding energy is a negative value, the inhibitor binds to the enzyme spontaneously without consuming energy; if the binding energy is a positive value that’s energy consuming. The elemolic acid (EA) showed a greater negative E value (− 309.23) than other molecules. Molecules like α-boswellic acid, lupeolic acid, and limonene also showed remarkable E values of − 282.35, − 280.46, and − 258.91, respectively (Table 1). Additionally, elemolic acid was reported for inhibitory activity against 12-O-tetradecanoyl phorbol-13-acetate-induced inflammation in mice^57^. If the elemolic acid inhibits inflammatory GIIA, that can also suppress inflammation by regulating the MAPK pathway^59^ is an added benefit.

**Table 1:** Energy values of molecules from *B. serrata* to bind GIIA (PDB id: 1POE).

SL. No	Compound name	Reference	PubMed ID	Molecular Formula	E value
1	α Boswellic acid	^60^	637234	C_30_H_48_O_3_	− 282.35
2	Cadinene	^61^	441005	C_15_H_24_	− 192.87
3	p-Cymene	^61^	7463	C_10_H_14_	− 186.78
4	Elemolic acid	^56,57^	441677	C_30_H_48_O_3_	− 309.23
5	Limonene	^62^	22311	C_10_H_16_	− 258.91
6	Lupeolic acid	^54,63^	12111950	C_30_H_48_O_3_	− 280.46
7	Phellendrene	^64^	7460	C_10_H_16_	− 197.25
8	Serratol	^56^	101618281	C_20_H_34_O	− 196.78
9	Genistein	Standard^65^	5280961	C_15_H_10_O_5_	− 286.47

**Figure 3:**
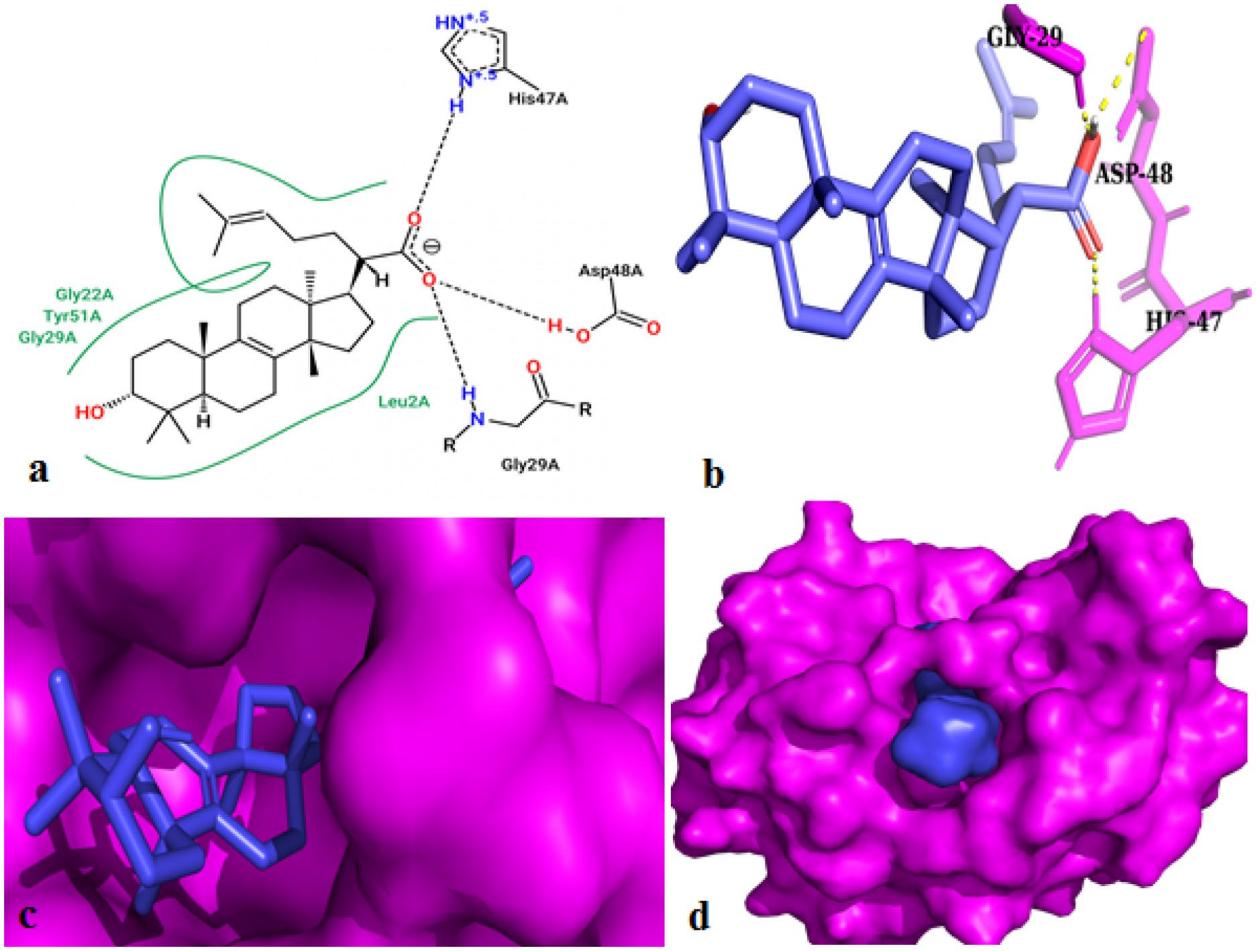
Docked images of GIIA (1POE) with elemolic acid. Stereoview of elemolic acid docked in the active site of GIIA. (**a**) Interaction between the active site amino acids of GIIA with elemolic acid and hydrophobic interaction with substrate-binding pocket; (**b**) Sticks display mode; (**c**, **d**) Space filled display modes were obtained from PyMol, Autodock Vina, and pose View (http://poseview.zbh.uni-hamburg.de/poseview). Hydrogen bonds are presented by the dashed line.

The GIIA enzyme consists of active site His-47/Asp-48 (1POE) diad (active site sequence Asp-Xxx-Cys-Cys-Xxx-Xxx-His-Asp), and calcium binding loop (loop sequence Xxx-Cys-Gly-Xxx-Gly-Gly) are important for the activity^66^. Most of the GIIA inhibitors such as aristolochic acid, ascorbic acid, palmitate, and p-BPB interfere with the catalytic site by binding with His-47/Asp-48 and weakening the Ca^2+^ coordination that lowers the catalytic activity of enzyme^67,68^. Many GIIA inhibitors, such as gallic acid, vannilic acid, syringic acid, and protocatechuic acid, interact with substrate binding pockets and avoid enzyme–substrate interaction by forming van der Waals contacts with amino acids Phe-23, Phe-5, Leu-31, and Leu-2^69^. Considering the above aspects, elemolic acid was docked against the GIIA enzyme. The GIIA containing three major alpha-helices located at amino acid sequences 0–17, 38–53, and 80–100 play a vital role in catalysis. The elemolic acid interacted with N terminus helices (almost), mainly to active site containing Gly-29, His-47, and Asp-48 through a hydrogen bond (H bond), as well as hydrophobic interaction with amino acids such as Leu-2, Gly-22, and Tyr-51 (Fig. 3a,b). The space-filling model confirmed the coverage of elemolic acid in the catalytic site of GIIA (Fig. 3c, d). Thus, elemolic acid established the interaction with both the catalytic site and substrate-binding pocket of the GIIA enzyme.

**Figure 4:**
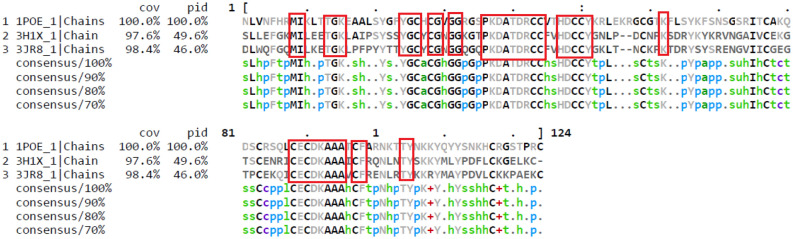
Sequence alignment of human GIIA (1POE), *B. jararacussu* GIIA (3JR8), and *V. russelii* GIIA (3H1X): Sequence of 1POE, 3JR8, and 3H1X were obtained from protein data bank and downloaded in fasta format. Alignment was done using Clustal Omega (https://www.ebi.ac.uk/Tools/msa/clustalo) and amino acid similarities visualized by MView (https://www.ebi.ac.uk/Tools/msa/mview).

There is a high degree of similarity between snake venom PLA_2_ and human GIIA, which share similar biological functions like acute muscle damage, pain, edema development, and leukocyte influx into tissues^70^. The structural homology and binding pattern of vannilic acid with human GIIA and *Bothrops jararacussu* Toxin II (BthTX-II), were very close to hydrogen bond energies, interaction energies and the score function^71^. *Bothrops jararacussu* and *Vipera russelii* are belongs to Viperidae family; their secreted enzymes, BthTX-II and *V. russelii* PLA_2_ (GIIA) are basic phospholipases. Hence, it is suggested that the use of *V. russelii* PLA_2_ (GIIA) as a tool for studying the mechanism of action and development of new inhibitors for the human GIIA enzyme^71^. Also, sequencing alignment of human GIIA (1POE), with *V. russelii* GIIA (3H1X) and *B. jararacussu* GIIA (3JR8) showed 97.6% and 98.4% homology, respectively, and 100% homology in active site residues (Fig. 4). Hence, in the present study, *V. russelii* PLA_2_ (GIIA) was considered for evaluating human GIIA inhibitor/s.

**Figure 5:**
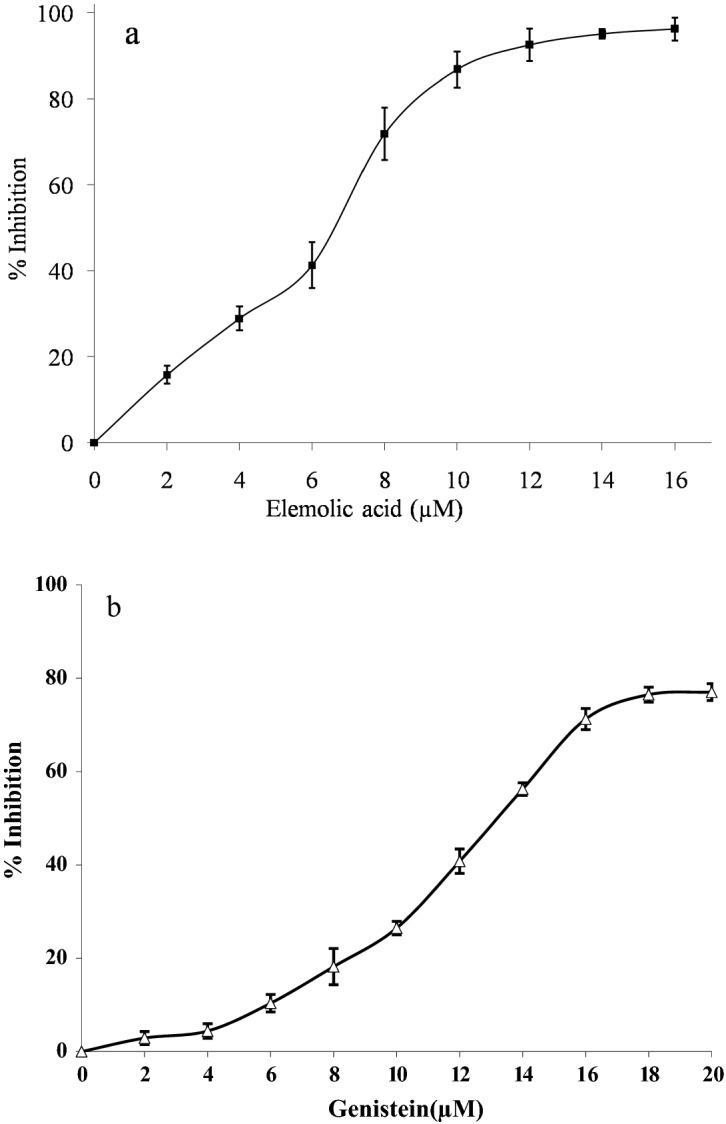
Inhibition of GIIA enzyme by elemolic acid (**a**) and genistein (**b**). Briefly, 350µL reaction mixture consists of 3.18 × 10^9^ autoclaved *E. coli* cells, 5 mM calcium, and 100 mM Tris–HCl buffer pH 7.4, with GIIA and indicated concentration of inhibitors, incubated at 37 °C for 60 min. GIIA activity was measured by the radiation of ^14^C using Quantulus 1220 liquid scintillation spectrometer (Perkin Elmer, USA). GIIA inhibition was noted as a percentage of control. The data are represents mean ± SD (n = 3).

Further, elemolic acid was subjected to inhibit GIIA enzyme, which showed concentration-dependent inhibition (Fig. 5). The extent of GIIA inhibition was 96% at 16 µM concentration with F-statistic value of 0.0035 and *p*-value 0.9965 (F static value is quantified additional errors of variances of the experimental data, this can be converted to probability value (*p*-value), and which is a statistical measure describe the probability of obtaining the observed results). Both the F-stat and *p*-values of GIIA inhibition by elemolic acid following the null hypothesis. The IC_50_ value of elemolic acid was 5.70 ± 0.02 µM, whereas the IC_50_ value of the positive control genistein was 11.92 ± 1.45 μM (Table 2)^65^.

**Table 2:** IC_50_ value of elemolic acid for GIIA inhibition.

Enzyme source	Specific activity* (nmol/mg/min at 37 °C)	IC_50_ (μM)^#^	
		Elemolic acid	Genistein
GIIA	146.0	5.70 ± 0.02	11.92 ± 1.45

*nmoles of fatty acid released/mg of protein/min at 37 °C.

^#^IC_50_ value is defined as the amount of inhibitors (µM) required to inhibit 50% of enzyme activity. The IC_50_ concentration was calculated using a Graph pad prism 5.0.

**Figure 6:**
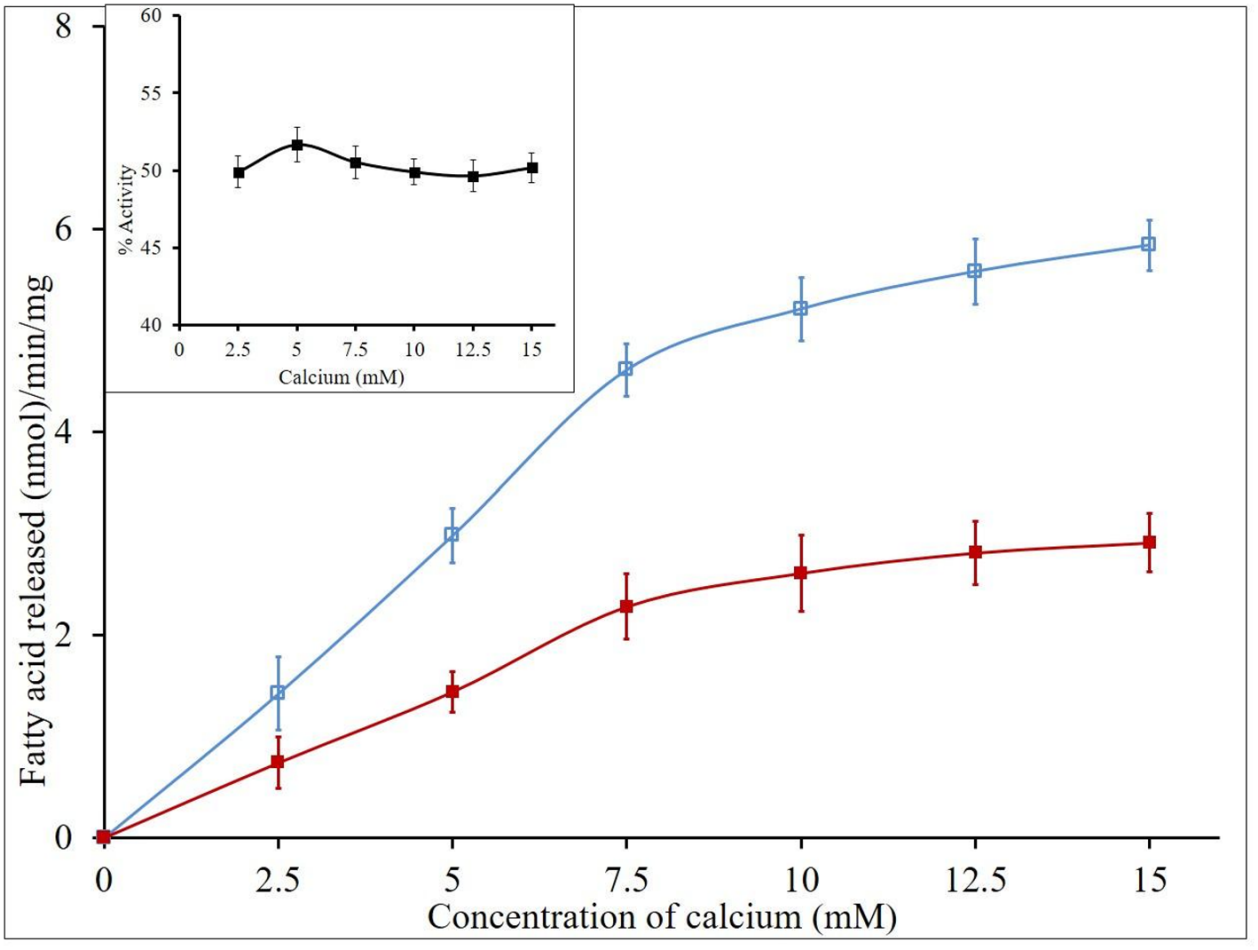
Effect of calcium concentration on inhibition of GIIA by elemolic acid. The reaction was performed with (red filled square) and without (blue open square) IC_50_ concentration of elemolic acid and increased the Ca^2+^ concentration from 2.5 to 15 mM. The GIIA inhibition was recorded in the presence of IC_50_ concentration inhibitor and has shown inlet. The data are expressed in mean ± standard deviation (n = 3).

**Figure 7:**
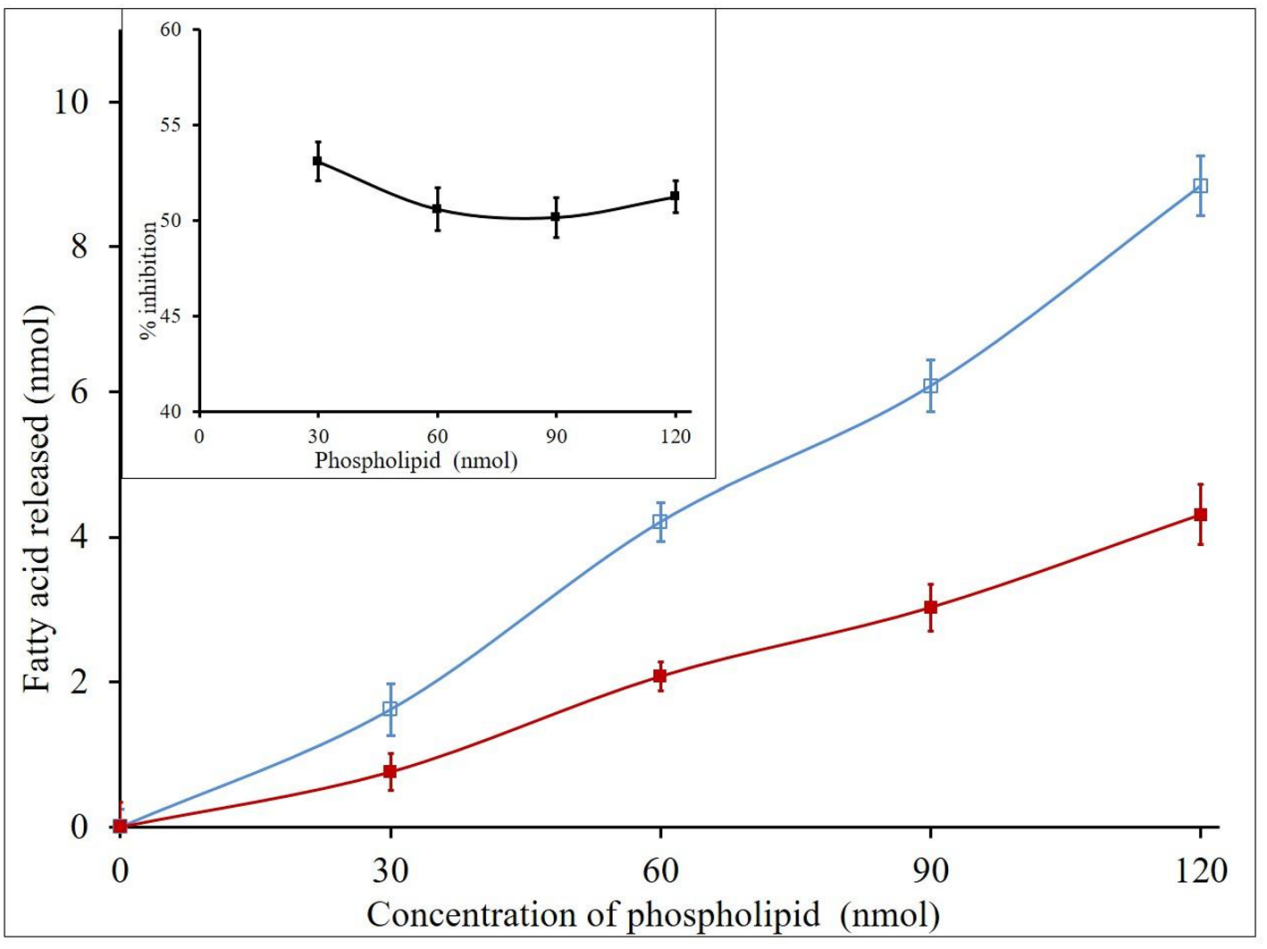
Effect of substrate concentration on inhibition of GIIA by elemolic acid: The reaction was performed with (red filled square) and without (blue open square) IC_50_ concentration of elemolic acid; the substrate concentration increased from 30 to 120 μL. The GIIA inhibition was recorded in the presence of IC_50_ concentration of elemolic acid and has shown inlet. The data are expressed in mean ± standard deviation (n = 3).

Because some of the inhibitors limit the activity of GIIA by either chelating metal ion calcium or some of the steroid inducible inhibitors (lipocortin I and II) non-specifically binding to GIIA impact the ‘quality of interface of phospholipids^72^. Hence, we investigated the effect of calcium and substrate concentrations on GIIA inhibition by elemolic acid. GIIA activity was measured in the presence and absence of IC_50_ concentration of elemolic acid (5.70 ± 0.02) by increasing the calcium concentration from 2.5 to 15 mM. The activity was increased linearly with constant inhibition, i.e., 49.2 ± 1.46% in all over the ranges of calcium concentrations (Fig. 6). Similarly, GIIA activity was measured by increasing the concentration of substrate from 30 to 120 nmoles, in the presence of IC_50_ concentration of elemolic acid; the activity was increased linearly and maintained constant inhibition i.e., 48.27 ± 1.38% in all over the ranges of substrate concentrations (Fig. 7). These results suggest that GIIA inhibition by elemolic acid is independent of calcium and substrate concentrations and does not alter enzyme activity by either binding to the substrate or chelating calcium ions.

**Figure 8:**
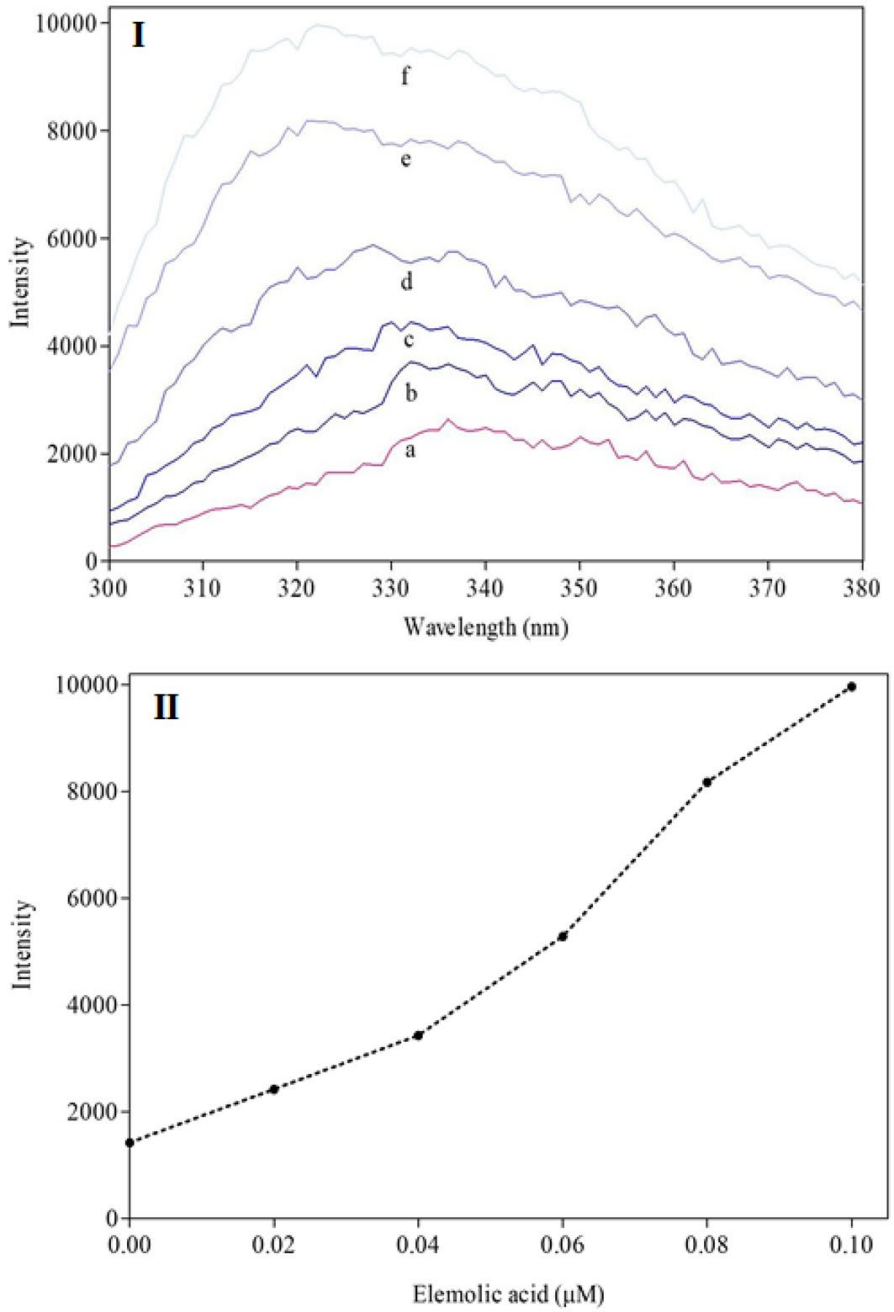
Intrinsic fluorescence spectra of GIIA enzyme with elemolic acid: (**I**) The spectra of (**a**) GIIA enzyme (20 µg/mL) alone, (**b**) with 0.02 µM, (**c**) 0.04 µM, (**d**) 0.06 µM, (**e**) 0.08 µM and (**f**) 0.1 µM of elemolic acid. (**II**) Maximum absorbance of fluorescence emission of GIIA enzyme after the addition of each concentration of elemolic acid was recorded.

Further, the intrinsic fluorescence of GIIA was measured to determine the changes in the structure of the enzyme in the presence of elemolic acid. The altered intrinsic fluorescence indicates the structural changes in the enzyme due to the interaction with the inhibitor. The interaction of many GIIA inhibitors with enzymes resulted in fluorescence quenching^73^. Generally, aromatic amino acids of proteins (tryptophan, tyrosine, and phenylalanine) contribute to the intrinsic fluorescence. The intensity, quantum yield, and wavelength of maximum fluorescence emission of these amino acids depend on the microenvironment of the amino acid molecules. The fluorescence spectrum shifts to a shorter wavelength, and the intensity of the fluorescence increases as the polarity of the solvent surrounding the aromatic amino acid residue decreases^74,75^. Elemolic acid alters the relative intrinsic fluorescence of GIIA in concentration dependent manner. The maximum intensity of GIIA was recorded at 338 nm and shifted towards a lower wavelength of 322 nm at 0.1 μM concentration of elemolic acid (Fig. 8I,II). GIIA contains aromatic amino acids such as Tryptophan 30; Tyrosine 21, 24, 27, 51, 64, 66, 103, 107, 110; Phenylalanine 45, 113 might be responsible for increased intrinsic fluorescence. Either elemolic acid or DMSO does not alter intrinsic fluorescence. Altered intrinsic fluorescence on the addition of elemolic acid indicates that the inhibitor interacts with the GIIA enzyme directly. Further, the dissociation constant (KD) of the elemolic acid was 6.805 ± 0.06 μM.

**Figure 9:**
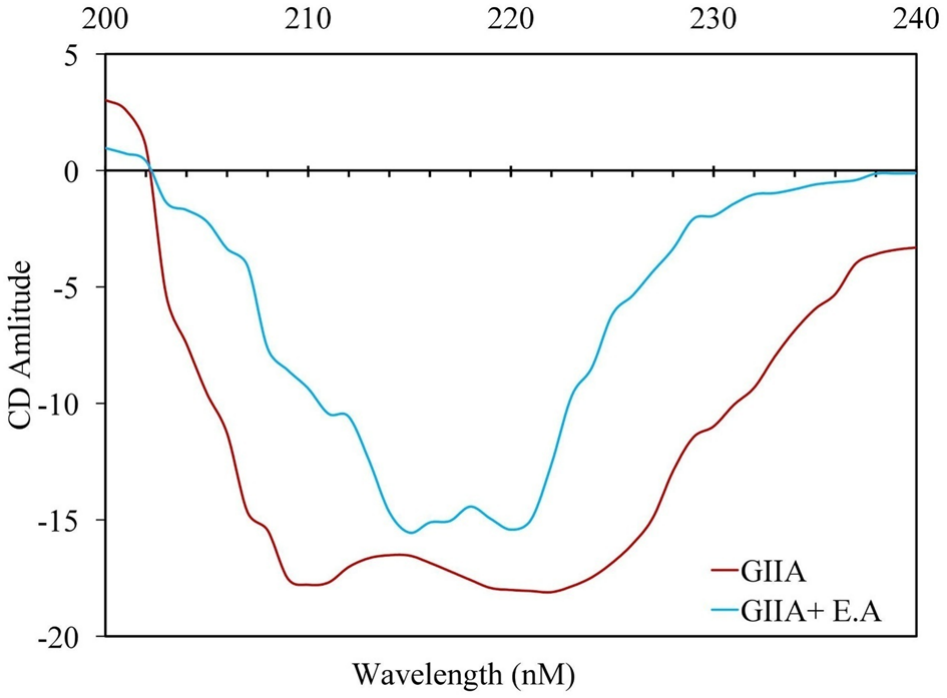
Far UV Circular Dichroism spectra of GIIA with and without elemolic acid. Far UV-CD spectra were recorded between 200 and 240 nm on a Jasco J715 spectropolarimeter. (**a**) UV-CD spectra of GIIA (30 µg/mL) alone, and (**b**) GIIA with IC_50_ concentration of elemolic acid.

A circular Dichroism experiment is commonly performed to analyze the structural changes in enzymes due to enzyme-inhibitor complex formation^76^. Generally, in CD analysis, the α-helix gives negative bands at 222 and 208 nm, β-sheet structures give a negative band at 210—220 nm, and the random coil has a characteristic negative band at 200 nm^77^. The far UV-CD spectrum of GIIA exhibited two distinct negative bands at 210 nm and 222 nm. The maximum absorbance of negative bands of GIIA was substantially reduced in the presence of elemolic acid at its IC_50_ concentration. The peak at 210 nm shifted abruptly towards a higher wavelength and formed a peak at 215 nm, and the peak at 222 nm shifted towards a lower wavelength and formed a peak at 220 nm (Fig. 9). The change in the secondary structure of GIIA upon the interaction of elemolic acid (IC_50_ concentration) was calculated using K2D3 software (Table 3). The changes in the CD spectrum of GIIA enzyme substantiate the findings of fluorimetric studies.

**Table 3:** Effect of elemolic acid on the secondary structure of GIIA.

Secondary structures	GIIA (%)	GIIA + elemolic acid (IC_50_) (%)
α-helix	48.31	22.14
β-sheet	13.71	29.7
Random coil	37.98	48.16

Further, the reversibility of GIIA inhibition was examined by subjecting the reaction mixture to dialysis. GIIA enzyme was pre-incubated with IC_50_ concentration of elemolic acid, and activity was checked before and after dialysis. The percentage inhibition before and after the dialysis was 50.4 ± 1.6 and 48.6 ± 1.7, respectively. This indicates that the elemolic acid binds to the GIIA irreversibly (Table 4).

**Table 4:** Inhibition of GIIA enzyme activity by elemolic acid before and after dialysis.

Reaction mixture	% of inhibition (Before dialysis)	% of inhibition (After dialysis)
GIIA + IC_50_ of elemolic acid	50.4 ± 1.6	48.6 ± 1.7

The data are expressed in mean ± standard deviation (n = 3).

**Figure 10:**
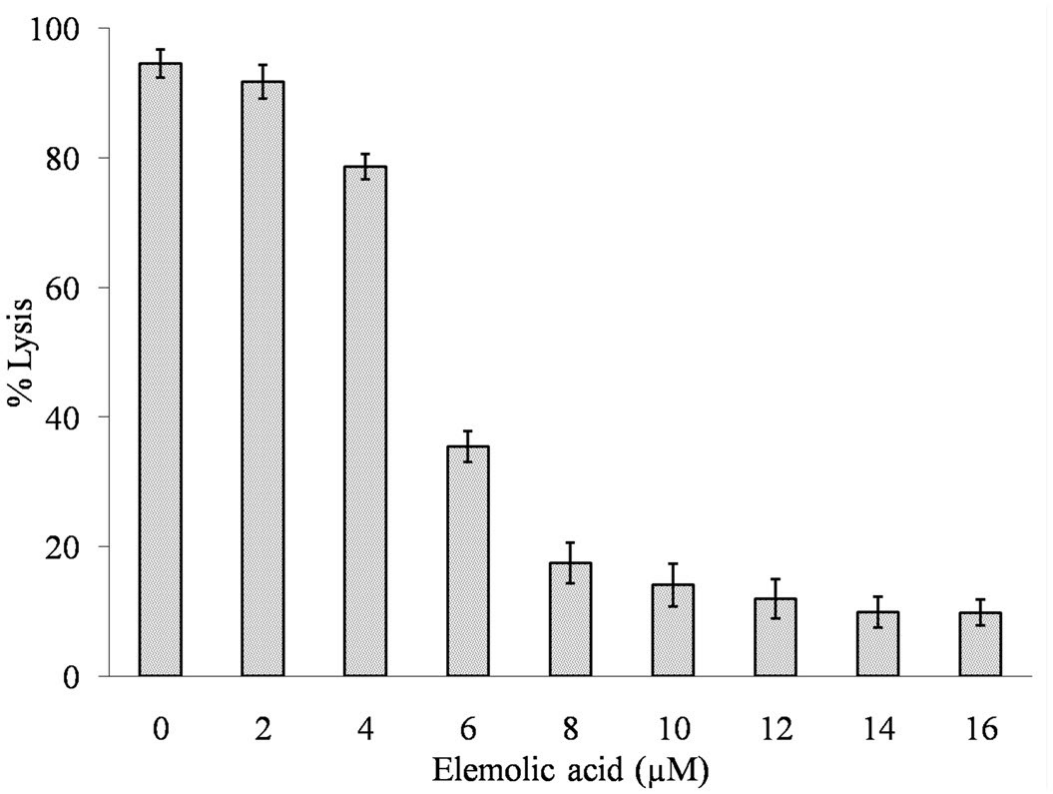
Neutralization of indirect hemolytic activity of GIIA by elemolic acid. The GIIA enzyme was pre-incubated with indicated concentrations of elemolic acid for 15 min. The reaction was initiated by adding 1 mL of the substrate (erythrocytes, egg yolk, and PBS -1: 1: 8 V/V) and incubated for 30 min at 37 °C. The hemoglobin released due to hemolysis was measured at 540 nm. The reaction mixture without enzymes served as a positive control. The data represents mean ± SD (n = 3).

The indirect hemolytic activity is an indirect approach to determine GIIA activity by using different substrates, i.e., egg yolk phospholipid and cleansed erythrocyte^78^. Elemolic acid (2 to 16 μM) was employed to neutralize the indirect hemolytic activity of GIIA, which neutralized the indirect hemolytic activity of GIIA in a concentration-dependent way. The GIIA (30 μg) alone caused the erythrocyte lysis to 94.5% ± 2.19 and which is reduced to 9.8% ± 2.39 at 16 μM elemolic acid (Fig. 10).

**Figure 11:**
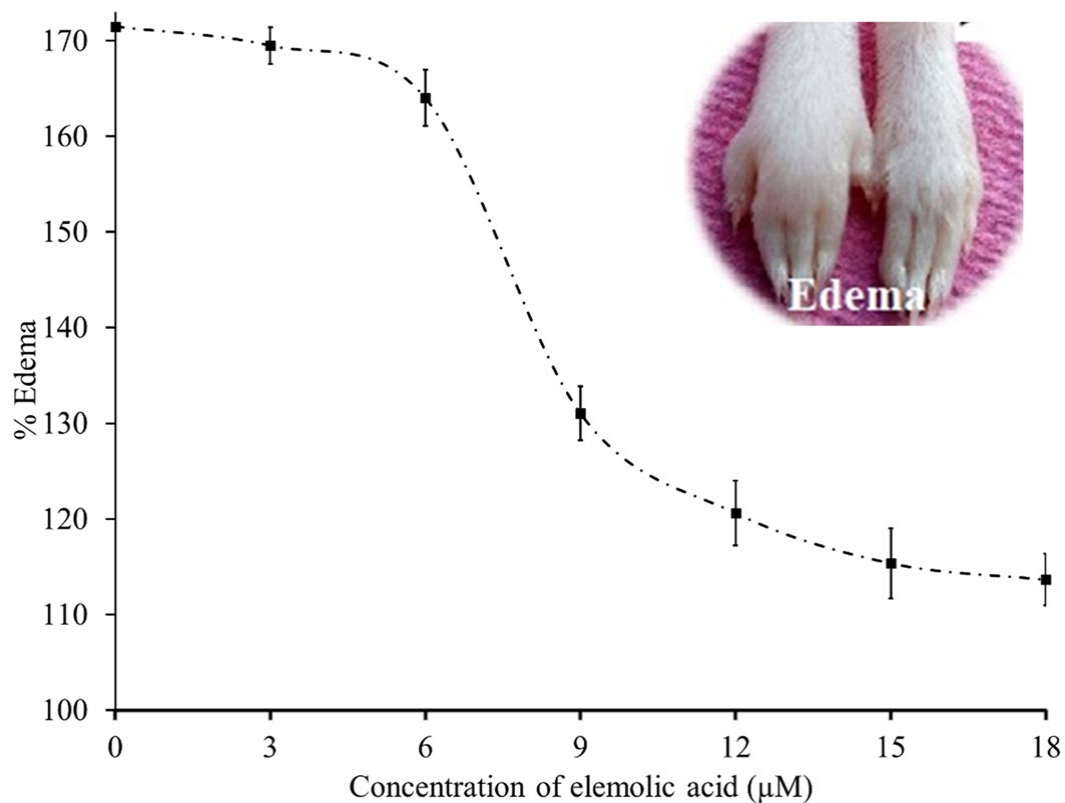
Neutralization of edema inducing activity of GIIA by elemolic acid. GIIA (5 µg) was pre-incubated with indicated concentration of elemolic acid (3 to 18 µM) for 30 min and injected into the right footpad of the hind limb of mice and the respective left footpad received vehicle (saline). After 45 min, mice were euthanized, and their legs were removed at the ankle joints and weighed separately. The edema ratio was calculated. The data are expressed in mean ± standard deviation (n = 3).

The ***p-***Bromophenacylbromide (*p*-BPB) neutralizes the phospholipase A_2_ (*V. russelii*) enzyme induced mouse paw edema^79^ by alkylating histidine-48, which is a highly conserved residue at the active site of *V. russelii* PLA_2_ (GIIA)^80,81^ and suggests that catalytic activity of GIIA is necessary to induce edema. Triterpenoids inhibitors of GIIA, such as celastrol, ursolic acid, oleanolic acid, neutralized GIIA induced mouse paw edema by binding to the catalytic domain of the enzyme. Hence, elemolic acid was tested for neutralizing GIIA induced edema. The different doses of elemolic acid were pre-incubated with GIIA and injected into the right hind paw of mice, and the left hind paw received saline as a negative control. Elemolic acid reduced the edema in a dose-dependent pattern and the edema ratio was reduced from 171.75% ± 2.39 (edematous leg) to 113.68% ± 2.74 at 18 μM concentration (Fig. 11). The apparent IC_50_ value of elemolic acid for reducing edema was found to be 7.98 μM. The edema ratio of the standard was 119% ± 2.20.

**Figure 12:**
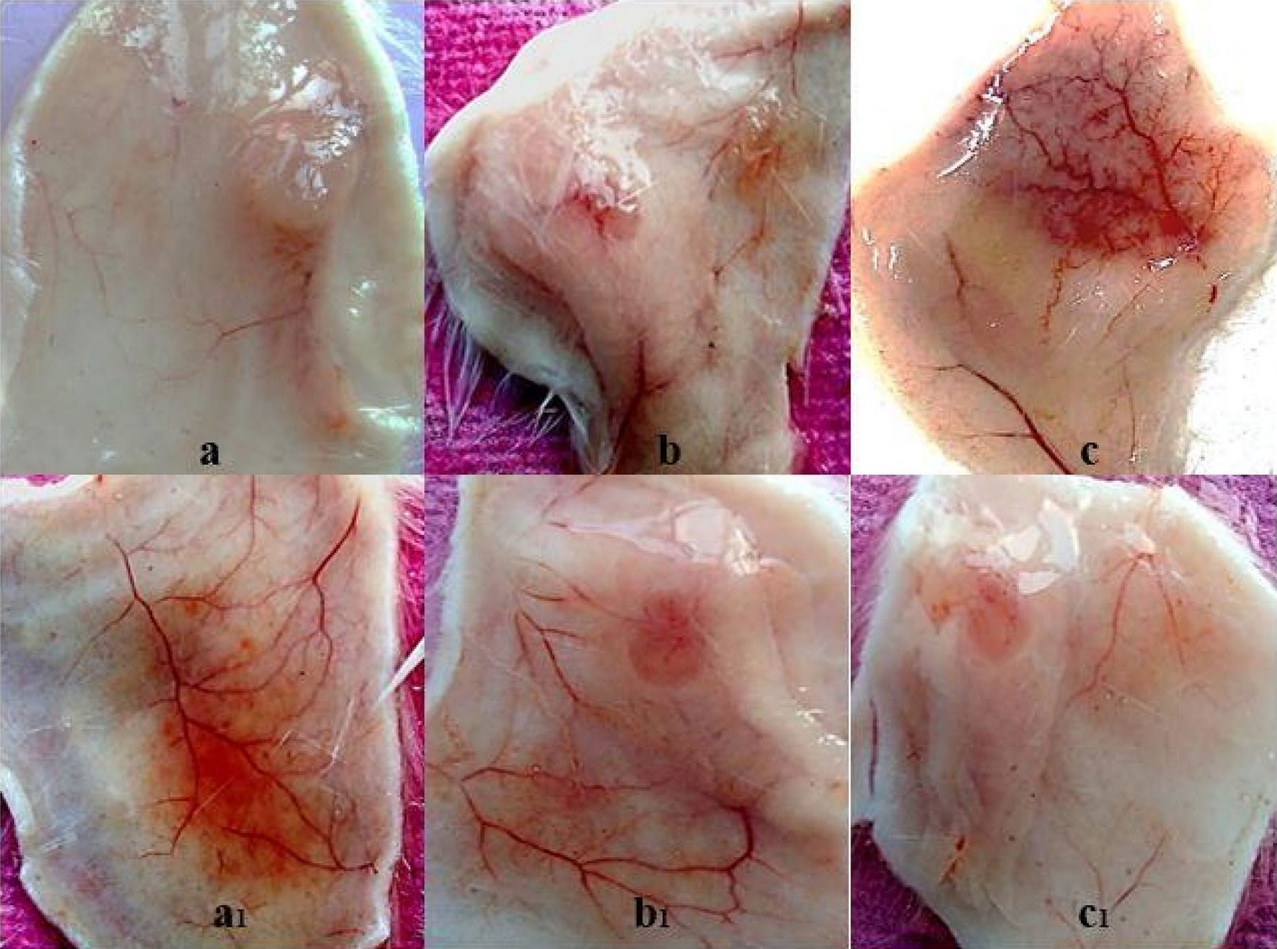
Inhibition of hemorrhagic activity of VR-HC-I by elemolic acid: Mice were intradermally injected the 10 µg of GIIA alone (**a**), 10 µg of non-enzymatic peptide (VNTx-II) alone (**b**), 10 µg of VR-HC-I (**c**); and 5 µM (**a**_**1**_), 10 µM (**b**_**1**_), 15 µM (**c**_**1**_) of elemolic acid was injected after 30 min incubation with 10 µg hemorrhagic complex (VR-HC-I). Mice were sacrificed after 3 h, and hemorrhagic spots on the dorsal surface of the skin were examined and measured using the graph sheet and represented as area (mm^2^).

In the living system, protein–protein interaction leads to protein complexes and is crucial for almost all aspects of cellular dynamics^82^, which enhances the pharmacological injuries by their synergistic action. Many human diseases are the result of abnormal protein–protein interactions^83^. For instance, the binding of human vimentin (an intracellular protein externalized during platelet activation) to GIIA enhances its catalytic activity^84^. It suggested that interaction GIIA-vimentin causes a more deleterious effect during inflammation. The addition of LY311727 (GIIA inhibitor which binds to the active site of enzyme) causes substantial structural changes in the amino terminus of the GIIA^85^. The structural displacement around the active site of GIIA by inhibitor is enough to reduce its interaction with vimentin. In snake bites, the synergistic interaction between GIIA and non-enzymatic peptides leads to increased hemorrhage^54^. In the current study, GIIA and *V. russelii* neurotoxic non-enzymatic peptide (VNTx-II) were used to form a protein–protein complex (5:2 molar ratio) called *V. russelii* Hemorrhagic Complex-I (VR-HC-I)^54^. Administration of the VR-HC-I complex into mice causes a synergistic hemorrhage at the injection site (Fig. 12c). On the other hand, either the GIIA or VNTx-II separately did not cause a hemorrhagic effect (Fig. 12a,b). Further, VR-HC-I was pre-incubated with different concentrations of elemolic acid (5 µM, 10 µM, and 15 μM) and administered to mice, resulting in the reduced hemorrhagic potential of VR-HC-I (Fig. 12a1, b1 and c1 respectively). After 30 min, mice were sacrificed, the skin was removed, the hemorrhagic spots on the dorsal surface were measured using a graph sheet, and the results were expressed in mm^2^. Elemolic acid significantly neutralized the hemorrhagic activity at 15 μM concentration.

In addition, the formation of oxidants to a higher extent signifies outrage of inflammatory response. Natural compounds that neutralize or reduce the generation of oxidants are known to have anti-inflammatory activity^86^. Hence, elemolic acid has been examined for its antioxidant activity in terms of its capacity to scavenge free radicals. At 25 µM concentration, elemolic acid efficiently scavenged the DPPH free radicals to 86.9% ± 2.3 compared to ascorbic acid, 98.5% ± 1.7. Elemolic acid showed a reducing power activity to 43.56% ± 1.97 compared to standard quercetin, which was 56.25% ± 2.5. The percentage of anti-lipid peroxidation activity of elemolic acid was 82% ± 2.2, whereas standard α-lipoic acid exhibited 89.6% ± 1.87 (Table 5). Thus, we can conclude that elemolic acid effectively scavenged the free radicals in all the three experimental methods.

**Table 5:** The in vitro antioxidant activities of elemolic acid.

Antioxidant activity	Elemolic acid (25 µM)	Vitamin C (25 µM)	Quercetin (25 µM)	α-Lipoic acid (25 µM)
DPPH free radical	86.90% ± 2.3	98.5% ± 1.70	NT	NT
Reducing power	43.56% ± 1.97	NT	56.25% ± 2.5	NT
Anti-lipid peroxidation	82.00% ± 2.2	NT	NT	89.6% ± 1.87

Antioxidant activities, DPPH free radical scavenging activity, reducing power activity, and anti-lipid peroxidation were tested for elemolic acid and compared with standards such as vitamin C, quercetin, α-lipoic acid, respectively.

NT, Not tested.

## Conclusion

The above data suggested that elemolic acid irreversibly binds to the GIIA and inhibits its activity proven by in vitro, in situ*, *in vivo studies. The studies on the mode of inhibition interpret that the inhibition of GIIA is not dependent on the concentration of metal ions or substrate. Altered intrinsic fluorescence and substantially reduced negative bands of CD spectrum by elemolic acid, indicating that the inhibitor interacted with GIIA enzyme directly. Also, elemolic acid neutralized the GIIA induced indirect hemolytic activity, mouse paw edema, and synergistic hemorrhagic effect (VNTx-II). Therefore, the elemolic acid is a candidate for drug development for both the inflammatory pathologies and snakebite envenomation. Further, necessary experiments are needed to prove elemolic acid as an anti-inflammatory drug.

## Data Availability

We declare that ‘The datasets used and/or analyzed during the current study available from the corresponding author on reasonable request’.
